# The motivational function of goals in physical activity: a cross-cultural comparison

**DOI:** 10.3389/fpsyg.2025.1642206

**Published:** 2026-01-06

**Authors:** Zitong Wang, Dominika Maria Wilczyńska, Ewelina Cichoń, Katarzyna Kreft, Junyu Lu, Małgorzata Lipowska, Ariadna Beata Lada-Masko, Bartosz M. Radtke, Urszula Sajewicz-Radtke, Bernadetta Izydorczyk, Anna Gmiąt, Paola Vago, Taofeng Liu, Mai Helmy, Violeta Enea, Negar Pourvakhshoori, Aidos Bolatov, Mariusz Lipowski

**Affiliations:** 1 Gdansk University of Physical Education and Sport, Gdańsk, Poland; 2 University WSB Merito Gdańsk, Gdańsk, Poland; 3 University of Lower Silesia, Wrocław, Poland; 4 University College of Professional Education in Wroclaw Poland, Wroclaw, Poland; 5 University of Gdańsk, Gdańsk, Poland; 6 Laboratory of Psychological and Educational Tests, Gdańsk, Poland; 7 Jagiellonian University, Kraków, Poland; 8 Catholic University of the Sacred Heart, Milano, Italy; 9 Zhengzhou University, Zhengzhou, China; 10 Sultan Qaboos University, Muscat, Oman; 11 Alexandru Ioan Cuza University, Iași, Romania; 12 Department of Nursing, School of Nursing and Midwifery, Guilan University of Medical Sciences, Rasht, Iran; 13 Human Research and Development LLP, Astana University, Astana, Kazakhstan

**Keywords:** exercise motivation, sport psychology, pro-healthy behavior, physical activity, nationality

## Abstract

**Objective:**

The aim of the study was to show the differences in the dominant motivational function of physical activity (PA) between women and men from Poland (PL), China (CN), Iran (IR), Kazakhstan (KZ), Egypt (EG), Romania (RO), and Italy (IT).

**Methods:**

A total of 6,195 participants (females = 3,376 and males = 2,819) aged 18–88 years (*M* = 28.5, SD = 12.74), residing in the seven countries, were analyzed for the purpose of this study. Twelve goal types were distinguished by the Inventory of Physical Activity Objectives (IPAO) tool were used in the study.

**Results:**

The analysis results indicated the significant main effects of gender and country. The three-way interaction between gender, type of goal, and country was significant, indicative that the largest differences in the rating of the goals’ importance between males and females occurred in evaluating objective 4 (fit, shapely body) in RO. Males in RO assessed this goal significantly lower than females. Among females, one of the largest differences occurred in the evaluation of the importance of objective 7 (boosting confidence, gaining appreciation from others) between PL and EG, as well as PL and IR.

**Conclusion:**

Understanding the motives behind PA and gender differences is crucial for developing effective health interventions and future cross-cultural research. The results of the study can be used in marketing by tailoring the content of the message to the audience. In schools, understanding the motives for physical activity will allow the curriculum to be adapted and thus increase student engagement in exercise. Understanding the profile of the exercise participant by the instructor will allow their needs to be effectively met and will positively correlate with persistence in activity.

## 1 Introduction

Motivation constitutes a central construct in understanding why individuals engage in, maintain, or abandon physical activity (PA). According to self-determination theory (SDT), the quality of motivation—ranging from autonomous to controlled regulation—shapes both the intensity and sustainability of PA behaviors (Deci and Ryan, 2000, 2002; Hagger and Chatzisarantis, 2008). Internal motivation, rooted in self-determination, personal satisfaction, and intrinsic enjoyment, yields long-term adherence and consistent performance improvement, whereas extrinsic forms—driven by rewards or social approval—tend to produce only short-term compliance (Alkasasbeh and Akroush, 2025; Deng et al., 2023; Kalajas-Tilga et al., 2020; Tock et al., 2024). The achievement goal theory (AGT) complements this perspective, emphasizing that mastery-oriented goals enhance persistence, enjoyment, and perceived competence, while performance-based goals may undermine sustained participation (Lochbaum and Gottardy, 2015; Lochbaum et al., 2013; Lochbaum et al., 2023; Mascret et al., 2015). In contrast, Zaleski's (1991) Motivational Goal Function theory highlights the key role of goals in human motivation. A goal is a realistic image of a future state of affairs that has subjective value for the individual and determines their behavior. This theory is based on the broader framework of goal-setting theory and emphasizes how goals, as cognitive motivators, energize and direct actions toward achieving desired outcomes. By providing direction and promoting effort, goals influence motivation and behavior, helping individuals achieve desired outcomes (Alispahić, 2013). Research indicates that both the goals people pursue and the reasons behind those goals significantly influence engagement in physical activity (Deci and Ryan, 2000; Lindwall et al., 2016). Understanding the goals people set for themselves provides an opportunity to understand their behavior. Therefore, the main research objective was explicitly defined as verifying cross-cultural and gender-related differences in the motivational goals underlying physical activity engagement among respondents from Poland (PL), China (CN), Iran (IR), Kazakhstan (KZ), Egypt (EG), Romania (RO), and Italy (IT). This objective stems from the broader aim of understanding how sociocultural and gender variables influence health-related behaviors and informs public health strategies promoting active lifestyles.

The study shows that motivational differences reflect different socialization patterns, cultural norms, and value systems prevailing in a given society. The identified differences are important for the design of health-promoting interventions and strategies for promoting physical activity. The results can be used in practice to develop personalized incentive programs that take into account local cultural conditions and gender differences in the perception of physical activity. In the context of health policy, this means that messages promoting activity need to be differentiated—in countries where health motives dominate, the prevention of lifestyle diseases and mental wellbeing should be emphasized, while in countries where competition is important, narratives related to achievement and success should be used. In addition, the data obtained can form the basis for developing educational strategies aimed at physical education teachers, coaches, and sports instructors who, knowing the motivational profile of participants, will be able to better adapt their working methods to individual needs. From a social perspective, the study contributes to a better understanding of how cultural values shape pro-health behaviors, which can support the development of sustainable public policies in the area of health.

In relation to the main aim of the study, the following section discusses separately the issue of gender and cultural affiliation as key factors determining motivational patterns regarding physical activity.

### 1.1 Gender as a factor determining the motives for physical activity – a review of studies

Empirical evidence across various cultural contexts consistently demonstrates that gender plays a significant role in shaping motivational patterns for engaging in physical activity. Studies conducted in Finland (Uimonen et al., 2021), the United States (Alecu et al., 2025), India (Salgaonkar and Mahadevan, 2025), Nigeria (Eo and In, 2024), as well as meta-analyses (Zhang et al., 2024), indicate that women more often declare internal motivation (pleasure, health), while men more often indicate external motivation (competition, appearance) for physical activity. A study conducted by Wilczyńska et al. (2021) showed that Polish women were characterized by a significantly higher escape from everyday life than Polish men, and Chinese women had a significantly higher level of social motivation than Chinese men. Furthermore, male students in China showed greater involvement in sports, driven by socialization and motivation to have fun, while female students prioritized aspects of physical appearance and health (Li and Pengfei, 2024). Other studies indicate that women exercised to lose weight and tone their bodies more often than men, while men exercised for pleasure more often than women (Craft et al., 2014). The reviewed literature supports the formulation of the first research question: whether gender differentiates motives for physical activity across the seven national groups under investigation. The next section expands this perspective by addressing cross-national variations in motivational structures.

### 1.2 Motivations for physical activity: a review of research from an international perspective

Cross-national research highlights substantial cultural variation in physical activity motivations. When examining global motivational trends, for instance in China, a study involving 2,544 students found health motivation to be the primary reason for engaging in PA, followed by motivations related to appearance, competence, and social factors (Liu et al., 2023). Another Chinese study highlighted that motivations differ based on the type of activity: health, stress management, and belongingness were key for physical exercise, whereas cultural leisure activities were driven by stress management, pleasure, and belongingness (Rahman et al., 2019). Another qualitative study on extreme leisure sports in China identified seven PA motives: mastery, enjoyment, physical fitness, belonging, mental fitness, expectations of others, and competition (Molanorouzi et al., 2015; Zhou et al., 2019). In Egypt and Saudi Arabia, medical students were motivated by health maintenance, weight control, muscle strength, and body image (El-Gilany and El-Masry, 2011). In Europe, for example, 93.2% of Romanian respondents cited health and fitness as their main PA motivators, followed by mental wellbeing, stress relief, appearance, and happiness (Buhaș et al., 2020). Research in Italy revealed that 67.6% of respondents cited health maintenance as their top PA motivator, similar to findings from Norway (Gjestvang et al., 2020). Additionally, the study on Ukrainian and Polish medical students revealed that Ukrainian students cited self-esteem improvement as their main PA motivator, with weight control being more prominent than among Polish students, despite lower obesity rates. Both Ukrainian and Polish respondents recognized the health benefits of PA (Kosendiak et al., 2023). According to a CBOS (2018) report, 69% of Poles engage in sports most often for health reasons, followed by pleasure (55%) and to improve their wellbeing and relieve stress (44%). Czarnecki et al. (2022) and his colleagues also showed that health is the main motivator among women and men in Poland. In turn, a report by the Association of Southeast Asian Nations (ASEAN, 2021) indicates that in all age groups (youth, adults, seniors), the main motivators for physical activity are physical and mental health, as well as good appearance and wellbeing. A study analyzing the motives for regular physical activity among medical students from the Western Balkans suggests that the most frequently declared motives are improved wellbeing, stress reduction, improved appearance, weight loss, and control of chronic diseases (Ilić et al., 2022). A profile of the motives for sports activity among elite athletes from Kazakhstan showed that the leading motivation was achievement motivation, followed by self-improvement and the need to compete (Krasmik et al., 2023). Consequently, the second research question sought to determine whether nationality differentiates the motivational goals associated with physical activity.

## 2 Materials and methods

### 2.1 Participants

There were seven countries included in this study: CN, EG, IR, IT, PL, KZ, and RO. The sample consisted of a total of 6,195 participants (54.5% female, 45.5% male), aged 18–88 years (*M* = 28.5, SD = 12.74), mostly single—never married (61.21%). Most participants live in the city, and only 27.64% live in the village. Regarding education level, most of the participants have a higher level of education (41.23%—bachelor’s degree, 15.77%—master’s degree, 3.52%—MD/PhD/doctorate). Above 48% (48.31) were students, and 30.57% were employed for wages. Detailed sociographic data by country are presented in Table 1.

**Table 1:** Characteristics of the study sample according to the country.

*N*		CN 1,467		EG 1,183		IR 699		IT 542		KZ 728		PL 1,084		RO 492		Total 6,195	
		*M*	SD	*M*	SD	*M*	SD	*M*	SD	*M*	SD	*M*	SD	*M*	SD	*M*	SD
		*n*	%	*n*	%	*n*	%	*n*	%	*n*	%	*n*	%	*n*	%	*n*	%
Age		26.04	8.83	19.72	1.61	32.07	10.95	27.92	12.54	26.22	10.75	38.38	17.55	34.02	11.69	28.50	12.74
Gender	Female	783	53.37	591	49.96	347	49.64	320	59.04	362	49.73	642	59.23	331	67.28	3,376	54.50
	Male	684	46.63	592	50.04	352	50.36	222	40.96	366	50.27	442	40.77	161	32.72	2,819	45.50
Place of residence	Village	276	18.81	839	70.92	30	4.29	212	39.11	148	20.33	126	11.62	81	16.46	1712	27.64
	Small town (up to 20,000 inh.)	328	22.36	109	9.21	61	8.73	70	12.92	46	6.32	76	7.01	72	14.63	762	12.30
	Medium town/city (20000–100,000 inh.)	313	21.34	164	13.86	150	21.46	136	25.09	68	9.34	141	13.01	108	21.95	1,080	17.43
	Big city (more than 100,000 inh.)	370	25.22	58	4.90	153	21.89	40	7.38	210	28.85	633	58.39	212	43.09	1,676	27.05
	Huge city (more than 1 million inh.)	180	12.27	13	1.10	305	43.63	84	15.50	256	35.16	108	9.96	19	3.86	965	15.58
The highest educational degree	Primary	30	2.04	1	0.08	0	0.00	2	0.37	132	18.13	18	1.66	5	1.02	188	3.03
	Vocational	36	2.45	0	0.00	2	0.29	2	0.37	48	6.59	30	2.77	15	3.05	133	2.15
	Secondary	162	11.04	987	83.43	155	22.17	164	30.26	222	30.49	350	32.29	85	17.28	2,125	34.30
	Bachelor’s degree	1,003	68.37	187	15.81	375	53.65	268	49.45	244	33.52	256	23.62	221	44.92	2,554	41.23
	Master’s degree	180	12.27	2	0.17	117	16.74	84	15.50	68	9.34	380	35.06	146	29.67	977	15.77
	MD/PhD/doctorate	56	3.82	6	0.51	50	7.15	22	4.06	14	1.92	50	4.61	20	4.07	218	3.52
Employment	Employed for wages	398	27.13	16	1.35	218	31.19	124	22.88	338	46.43	505	46.59	295	59.96	1894	30.57
	Self-employed	108	7.36	8	0.68	150	21.46	30	5.54	34	4.67	155	14.30	40	8.13	525	8.47
	Out of work and looking for work	36	2.45	3	0.25	73	10.44	14	2.58	34	4.67	57	5.26	37	7.52	254	4.10
	Out of work but not currently looking for work	46	3.14	3	0.25	12	1.72	14	2.58	28	3.85	60	5.54	8	1.63	171	2.76
	A homemaker	30	2.04	1	0.08	56	8.01	6	1.11	6	0.82	20	1.85	7	1.42	126	2.03
	A student	843	57.46	1,152	97.38	165	23.61	344	63.47	286	39.29	114	10.52	89	18.09	2,993	48.31
	Retired	4	0.27	0	0.00	17	2.43	6	1.11	2	0.27	169	15.59	9	1.83	207	3.34
	Unable to work	2	0.14	0	0.00	4	0.57	0	0.00	0	0.00	2	0.18	2	0.41	10	0.16
	Military	0	0.00	0	0.00	4	0.57	4	0.74	0	0.00	2	0.18	5	1.02	15	0.24
Marital status	Single, never married	1,178	80.30	595	50.30	494	70.67	469	86.53	504	69.23	332	30.63	220	44.72	3,792	61.21
	Married	250	17.04	327	27.64	172	24.61	58	10.70	182	25.00	383	35.33	129	26.22	1,501	24.23
	Domestic partnership	2	0.14	93	7.86	7	1.00	2	0.37	5	0.69	133	12.27	39	7.93	281	4.54
	Living apart together (LAT)	13	0.89	142	12.00	25	3.58	13	2.40	25	3.43	115	10.61	72	14.63	405	6.54
	Widowed	14	0.95	2	0.17	0	0.00	0	0.00	2	0.27	55	5.07	6	1.22	79	1.28
	Divorced	4	0.27	18	1.52	1	0.14	0	0.00	6	0.82	60	5.54	22	4.47	111	1.79
	Separated	6	0.41	6	0.51	0	0.00	0	0.00	4	0.55	6	0.55	4	0.81	26	0.42

### 2.2 Methods

The Inventory of Physical Activity Objectives (IPAO), developed by Lipowski and Zaleski (2015), was used in the study conducted. The IPAO is used to measure the motivational function of physical activity goals and allows for controlling variables such as the frequency of PA (understood as physical recreation activities or sports requiring physical effort that are undertaken in a person’s free time), the variety of forms, and socio-demographic conditions in competitive sport (both current and past), as well as their passive attitude. For the purpose of this study, we analyzed only the part of the IPAO that estimates the level of 12 physical activity goals. Respondents indicated to what extent the presented physical activity goals were important to them using a five-point Likert scale from 1 (completely unimportant) to 5 (very important). The 12 goals identified in the IPAO are as follows: Health (right levels of blood pressure, cholesterol, body mass, etc.); physical fitness, being ‘in shape’; company of other people; fit, shapely body (beauty, sculpted, and firm body); wellbeing; being physically active and fit according to fashion; boosting confidence, gaining appreciation from others; pleasure from physical activity; escape from everyday life; managing stress; fulfilling the need for activity; and promoting physical activity by setting a behavioral example.

### 2.3 Statistical analysis

To examine how the importance of the goals is influenced by the type of goal, gender, and country, we performed three-way mixed ANOVA with one within-subjects factor (12 types of goals were distinguished by the IPAO) and two between-subjects (grouping) factors: gender (male vs. female) and country (seven countries: CN, EG, IR, IT, PL, KZ, RO). The Games–Howell test was used for post-hoc comparisons for between-subject comparisons (country) as it is recommended to control for type I error per comparison in heterogeneous variances and unequal sample sizes (Agbangba et al., 2024; Toothaker, 1993). Bonferroni correction as an adjustment for multiple comparisons was used if significant within-subjects and interaction effects were observed. Post-hoc power analysis was conducted using G*Power (Cohen, 1988) to determine the statistical power for detecting the interaction effect in a repeated measures ANOVA. The analysis was based on a total sample size of 6,195, with eight groups and twelve repeated measures. The effect size was set to f(U) = 0.084, with an alpha level of 0.05. The results indicated that the statistical power (1 − *β*) for detecting the interaction effect was 1.00, suggesting that the study was well powered to detect even small effects. According to Cohen (Faul et al., 2007), power levels above 0.80 are considered adequate to detect true effects, reinforcing the robustness of the current study’s design. The statistical analyses were carried out using SPSS version 29 for Windows (IBM Corp, 2023). Statistical significance for all the conducted analyses was established at *p* < 0.05. Missing data were marked with the code 999 and declared in SPSS as user-defined missing values. Due to the small percentage of missing data, no imputation was used; these values were technically excluded from the analyses, which allowed the integrity of the data set to be preserved.

### 2.4 Procedure of the study

Participants were recruited for the survey through a multi-faceted approach to ensure diverse representation. Email invitations were sent to schools, universities, and cultural centers within various city and village areas across seven countries. Additionally, paper notices were placed in these institutions, including churches. Online platforms, such as social media, forums, and community boards, were utilized to promote the survey. QR codes in promotional materials provided easy access. Community events, virtual information sessions, SMS invitations, and a referral program further encouraged participation.

The inclusion criterion for the study was holding citizenship and residing in one of the seven surveyed countries. The survey used an online questionnaire, and participants completed the informed consent form before starting the survey.

## 3 Results

### 3.1 Main and two-way interaction effects

The analysis results indicated significant main effects of gender, *F*(1, 4,204) = 16.26; *p* < 0.001; η_p_^2^ = 0.004; and country, *F*(6, 4,204) = 25.65; *p* < 0.001; η_p_^2^ = 0.035. Both effects were small. The main effect of type of goal significantly violated the sphericity assumption (*W* = 0.25, *p* < 0.001). Therefore, the *F*-values for the main effect of goal and its interaction with the between-group variables (gender and country) were corrected for this violation using the Greenhouse–Geisser correction. The main effect of the type of goal was significant (*F*(8.725, 36678.407) = 574.65; *p_G-G_* < 0.001; η_p_^2^ = 0.120) (Table 2). The ringed interaction effect was moderate.

**Table 2:** Means, standard deviations, and results of 3-way mixed ANOVA for gender, country (between-subjects factors), and type of goals (within-subjects factor) as independent variables and importance of the goal as dependent variable.

Type of goal		CN	EG	IR	IT	KZ	PL	RO	Total
		*M* (SD)	*M* (SD)	*M* (SD)	*M* (SD)	*M* (SD)	*M* (SD)	*M* (SD)	*M* (SD)
Goal 1 Health (right levels of: blood pressure, cholesterol, body mass, etc.)	Fe	4.46 (0.92)	4.27 (1.02)	4.51 (0.89)	4.46 (0.83)	4.49 (0.86)	4.54 (0.76)	4.39 (0.86)	4.46 (0.88)
	Ma	4.47 (0.87)	4.31 (0.92)	4.53 (0.77)	4.54 (0.65)	4.27 (0.99)	4.18 (0.98)	4.14 (1.21)	4.35 (0.92)
	Total	4.46 (0.90)	4.30 (0.96)	4.52 (0.83)	4.49 (0.76)	4.36 (0.94)	4.39 (0.88)	4.30 (1.00)	4.41 (0.90)
Goal 2 Physical fitness, being ‘in shape’	Fe	4.25 (1.06)	4.23 (1.02)	4.65 (0.73)	4.49 (0.73)	4.33 (0.96)	4.58 (0.67)	4.34 (0.79)	4.41 (0.89)
	Ma	4.33 (1.02)	3.99 (1.20)	4.29 (0.92)	4.50 (0.65)	4.35 (0.98)	4.40 (0.87)	4.12 (1.10)	4.28 (1.01)
	Total	4.28 (1.04)	4.09 (1.13)	4.47 (0.85)	4.49 (0.70)	4.35 (0.97)	4.51 (0.76)	4.26 (0.91)	4.35 (0.95)
Goal 3 Company of other people	Fe	4.01 (1.07)	3.86 (1.15)	3.71 (1.28)	3.74 (1.22)	3.47 (1.29)	3.79 (1.20)	3.63 (1.24)	3.80 (1.19)
	Ma	3.72 (1.22)	3.92 (0.96)	3.66 (1.12)	3.93 (1.14)	3.84 (1.29)	3.57 (1.30)	3.36 (1.24)	3.75 (1.19)
	Total	3.89 (1.14)	3.90 (1.04)	3.69 (1.20)	3.82 (1.19)	3.70 (1.30)	3.71 (1.24)	3.54 (1.24)	3.77 (1.19)
Goal 4 Fit, shapely body (beauty, sculpted and firm body)	Fe	4.37 (0.90)	4.32 (0.96)	4.65 (0.71)	4.05 (0.95)	4.39 (0.91)	4.28 (0.93)	4.00 (1.06)	4.30 (0.94)
	Ma	4.32 (0.92)	4.33 (0.81)	4.29 (0.81)	3.93 (1.06)	4.14 (1.10)	3.84 (1.10)	3.21 (1.24)	4.11 (1.02)
	Total	4.35 (0.91)	4.33 (0.87)	4.47 (0.78)	4.00 (1.00)	4.24 (1.03)	4.11 (1.02)	3.72 (1.18)	4.21 (0.98)
Goal 5 Wellbeing	Fe	4.45 (0.86)	4.28 (1.00)	4.79 (0.53)	4.80 (0.52)	4.59 (0.84)	4.73 (0.55)	4.53 (0.70)	4.59 (0.76)
	Ma	4.53 (0.79)	4.21 (0.97)	4.61 (0.78)	4.79 (0.48)	4.48 (0.87)	4.49 (0.84)	4.35 (0.87)	4.47 (0.85)
	Total	4.48 (0.83)	4.24 (0.98)	4.70 (0.67)	4.80 (0.50)	4.52 (0.86)	4.63 (0.69)	4.47 (0.77)	4.53 (0.80)
Goal 6 Being physically active and fit according to fashion	Fe	3.46 (1.32)	4.03 (1.11)	4.39 (0.89)	3.27 (1.32)	3.73 (1.30)	3.02 (1.39)	3.44 (1.22)	3.53 (1.33)
	Ma	3.43 (1.32)	4.22 (0.94)	4.19 (1.00)	3.78 (1.31)	3.86 (1.26)	2.46 (1.41)	3.04 (1.34)	3.59 (1.37)
	Total	3.45 (1.32)	4.15 (1.01)	4.29 (0.95)	3.48 (1.34)	3.80 (1.28)	2.79 (1.42)	3.30 (1.27)	3.56 (1.35)
Goal 7 Boosting confidence, gaining appreciation from others	Fe	4.00 (1.12)	4.38 (0.96)	4.35 (1.03)	3.68 (1.13)	3.98 (1.17)	3.04 (1.49)	3.78 (1.16)	3.81 (1.28)
	Ma	3.97 (1.04)	4.35 (0.84)	4.00 (1.06)	3.86 (1.15)	3.95 (1.24)	2.74 (1.32)	3.41 (1.44)	3.80 (1.24)
	Total	3.99 (1.09)	4.36 (0.89)	4.18 (1.06)	3.76 (1.14)	3.96 (1.21)	2.92 (1.43)	3.65 (1.28)	3.80 (1.26)
Goal 8 Pleasure from physical activity	Fe	4.40 (0.87)	4.23 (1.01)	4.54 (0.82)	4.48 (0.89)	4.26 (1.04)	4.46 (0.83)	3.91 (1.03)	4.36 (0.92)
	Ma	4.36 (0.86)	4.20 (0.95)	4.39 (0.83)	4.54 (0.82)	4.33 (0.97)	4.33 (0.95)	4.16 (1.00)	4.33 (0.92)
	Total	4.39 (0.87)	4.21 (0.98)	4.47 (0.82)	4.51 (0.86)	4.30 (1.00)	4.41 (0.88)	4.00 (1.02)	4.34 (0.92)
Goal 9 Escape from everyday life	Fe	3.58 (1.19)	3.53 (1.26)	3.31 (1.46)	3.78 (1.24)	2.59 (1.40)	3.59 (1.16)	3.83 (1.13)	3.51 (1.28)
	Ma	3.67 (1.16)	3.38 (1.24)	3.24 (1.35)	3.79 (1.29)	2.67 (1.44)	3.80 (1.17)	3.67 (1.11)	3.44 (1.31)
	Total	3.62 (1.18)	3.44 (1.25)	3.27 (1.4)	3.79 (1.25)	2.63 (1.43)	3.67 (1.17)	3.78 (1.12)	3.48 (1.29)
Goal 10 Managing stress	Fe	4.09 (1.09)	4.02 (1.07)	4.36 (0.97)	4.25 (0.98)	4.20 (1.07)	4.03 (0.99)	4.21 (0.97)	4.14 (1.03)
	Ma	4.24 (0.91)	3.98 (1.08)	4.17 (0.98)	4.18 (0.9)	4.22 (1.04)	3.90 (1.24)	3.87 (1.22)	4.10 (1.06)
	Total	4.16 (1.02)	4.00 (1.07)	4.26 (0.98)	4.22 (0.94)	4.22 (1.05)	3.98 (1.10)	4.09 (1.07)	4.12 (1.04)
Goal 11 Fulfilling the need for activity	Fe	4.15 (0.95)	4.16 (0.96)	4.39 (0.86)	4.32 (0.88)	4.39 (0.99)	4.27 (0.91)	4.05 (0.94)	4.23 (0.93)
	Ma	4.13 (0.95)	4.05 (1.01)	4.19 (0.91)	4.27 (0.91)	4.28 (0.95)	3.99 (1.08)	3.92 (1.10)	4.12 (0.99)
	Total	4.14 (0.95)	4.09 (0.99)	4.29 (0.89)	4.30 (0.89)	4.33 (0.96)	4.16 (0.99)	4.00 (1.00)	4.18 (0.96)
Goal 12 Promoting PA by setting a behavior example	Fe	3.76 (1.10)	4.13 (0.97)	4.25 (0.98)	3.88 (1.29)	3.90 (1.24)	3.53 (1.25)	3.51 (1.12)	3.81 (1.17)
	Ma	3.75 (1.08)	4.19 (0.91)	4.14 (0.89)	4.26 (0.93)	4.01 (1.17)	3.24 (1.42)	3.36 (1.31)	3.86 (1.16)
	Total	3.76 (1.09)	4.16 (0.93)	4.19 (0.94)	4.04 (1.17)	3.97 (1.2)	3.41 (1.32)	3.46 (1.19)	3.83 (1.17)
Main effects:									
Gender	*F*(1, 4,204) *_=_* 16.26; *p* < 0.001; η_p_^2^ *_=_* 0.004								
Country	*F*(6, 4,204) *_=_* 25.65; *p* < 0.001; η_p_^2^ *_=_* 0.035								
Goal	*F*(8.725, 36678.407) *_=_* 574.65; *p_G-G_* < 0.001; η_p_^2^ *_=_* 0.120								
2-way interactions									
Gender x Country	*F*(6, 4,204) *_=_* 6.14; *p* < 0.001; η_p_^2^ *_=_* 0.009								
Gender x Goal	*F*(8.725, 36678.407) *_=_* 7.98; *p_G-G_* < 0.001; η_p_^2^ *_=_* 0.002								
Country x Goal	*F*(52.348, 36678.407) *_=_* 56.54; *p_G-G_* < 0.001; η_p_^2^ *_=_* 0.075								
3-way interactions									
Goal x Gender x Country	*F*(52.348, 36678.407) *_=_* 4.61; *p_G-G_* < 0.001; η_p_^2^ *_=_* 0.007								

CN, China; EG, Egypt; IR, Iran; IT, Italy; KZ, Kazakhstan; PL, Poland; RO, Romania; Fe, females; Ma, males.

It also turned out that the two-way interaction effects of gender x country, as well as goal x gender and goal x country, were significant (Table 2). However, due to the significant three-way interaction effects, the interpretation of the main effects and two-way interactions may be incomplete or misleading. Therefore, the interpretation of three-way interactions was further analyzed and interpreted.

### 3.2 Three-way interaction effects

Since the three-way interaction between gender, type of goal, and country was significant, *F*(52.348, 36678.407) = 4.61; *p_G-G_* < 0.001; η_p_^2^ = 0.007 (Table 2). The effect of the interaction of the three factors was small. We analyzed the two-way interaction (gender × type of goal) at each level of the third variable (country). We also repeated the procedure for the type of goal x country interaction at different levels of gender.

### 3.3 Gender x type of goal interaction at each level of country

The pairwise multiple comparisons (PMC) with Bonferroni correction (Table 3) indicated that the largest differences in the rating of the goals’ importance between males and females occurred in evaluating objective 4 (fit, shapely body) in RO. Males in RO assessed this goal significantly lower (*M* = 3.21; SD = 1.24) than females (*M* = 4.00; SD = 1.06). A high gender difference was also shown in the evaluation of goal 6 (being physically active and fit according to fashion) in PL (*p* < 0.001) and IT (*p* < 0.001). Females in PL assessed objective 6 as more important (*M* = 3.02; SD = 1.39) than Polish males (*M* = 2.46; SD = 1.41). In contrast, Italian females rated this target significantly lower (*M* = 3.27; SD = 1.32) than Italian males (*M* = 3.78; SD = 1.31).

**Table 3:** The results of multiple comparisons: significance of differences between gender (used as grouping factor) among types of goals in goal importance (dependent variable) in individual countries.

Country	Goal	Difference in means (Female–Male)	SE	*p_bonf_*
CN	1	−0.02	0.06	0.761
	2	−0.08	0.06	0.202
	3	0.29*	0.08	<0.001
	4	0.05	0.06	0.458
	5	−0.08	0.05	0.128
	6	0.03	0.08	0.720
	7	0.03	0.08	0.740
	8	0.04	0.06	0.514
	9	−0.09	0.08	0.287
	10	−0.15*	0.07	0.027
	11	0.01	0.06	0.833
	12	0.01	0.07	0.856
EG	1	−0.04	0.07	0.599
	2	0.24***	0.07	0.001
	3	−0.06	0.09	0.518
	4	−0.01	0.08	0.900
	5	0.07	0.06	0.268
	6	−0.20*	0.10	0.048
	7	0.04	0.09	0.708
	8	0.03	0.07	0.633
	9	0.15	0.10	0.127
	10	0.03	0.08	0.680
	11	0.11	0.08	0.166
	12	−0.06	0.09	0.507
IR	1	−0.01	0.08	0.897
	2	0.37***	0.09	<0.001
	3	0.05	0.11	0.632
	4	0.36***	0.09	<0.001
	5	0.18*	0.07	0.011
	6	0.20	0.11	0.078
	7	0.35***	0.11	0.001
	8	0.15	0.08	0.073
	9	0.08	0.12	0.514
	10	0.19*	0.10	0.048
	11	0.20*	0.09	0.022
	12	0.11	0.10	0.274
IT	1	−0.09	0.09	0.317
	2	−0.01	0.09	0.897
	3	−0.19	0.11	0.095
	4	0.12	0.09	0.192
	5	0.01	0.08	0.948
	6	−0.51***	0.12	<0.001
	7	−0.18	0.11	0.116
	8	−0.06	0.09	0.474
	9	−0.01	0.12	0.930
	10	0.06	0.10	0.528
	11	0.05	0.09	0.617
	12	−0.38***	0.11	0.001
KZ	1	0.23**	0.08	0.005
	2	−0.02	0.09	0.812
	3	−0.37***	0.11	0.001
	4	0.25**	0.09	0.004
	5	0.11	0.07	0.128
	6	−0.13	0.11	0.256
	7	0.03	0.11	0.798
	8	−0.07	0.08	0.398
	9	−0.08	0.12	0.481
	10	−0.02	0.10	0.814
	11	0.12	0.09	0.190
	12	−0.12	0.10	0.269
PL	1	0.36***	0.06	<0.001
	2	0.18**	0.07	0.005
	3	0.22**	0.08	0.009
	4	0.44***	0.07	<0.001
	5	0.24***	0.06	<0.001
	6	0.56***	0.09	<0.001
	7	0.30***	0.08	<0.001
	8	0.13*	0.07	0.048
	9	−0.21*	0.09	0.020
	10	0.13	0.07	0.079
	11	0.29***	0.07	<0.001
	12	0.29***	0.08	<0.001
RO	1	0.25*	0.10	0.015
	2	0.23*	0.11	0.033
	3	0.27*	0.14	0.047
	4	0.79***	0.11	<0.001
	5	0.18*	0.09	0.042
	6	0.40**	0.14	0.005
	7	0.37**	0.13	0.006
	8	−0.25*	0.10	0.016
	9	0.16	0.14	0.259
	10	0.33**	0.12	0.005
	11	0.13	0.11	0.234
	12	0.15	0.13	0.257

Only significant results were presented at the table. Goal: 1 – health (right levels of: blood pressure. Cholesterol. body mass. Etc.); 2 – physical fitness. Being ‘in shape’; 3 – company of other people; 4 – fit. Shapely body (beauty. Sculpted and firm body); 5 – wellbeing; 6 – being physically active and fit according to fashion; 7 – boosting confidence. Gaining appreciation from others; 8 – pleasure from physical activity; 9 – escape from everyday life; 10 – managing stress; 11 – fulfilling the need for activity; 12 – promoting pa by setting a behavior example.

p_bonf_ - *p*-value with Bonferroni correction.

^***^, p_bonf_ < 0.001; ^**^, p_bonf_ < 0.01; ^*^, p_bonf_ < 0.05.

In particular, the PMC showed that:

- In CN, females rated goal 3 (company of other people) significantly higher than male, and goal 10 (managing stress) significantly lower.
- In EG, females rated goal 2 (physical fitness) significantly higher than male, and target 6 (being physically active and fit according to fashion) was assessed significantly lower by females than by males.
- In IR, females rated targets 2 (physical fitness), 4 (fit, shapely body), 5 (wellbeing), 7 (confidence, appreciation), 10 (managing stress), and 11 (need for activity) higher than males.
- In IT, females assessed objectives 6 (being physically active and fit according to fashion) and 12 (promoting PA) significantly lower than males.
- In KZ, goals 1 (health) and 4 (fit, shapely body) were assessed significantly higher females than by males, and goal 3 (company of other people) was rated significantly lower by females.
- In PL, the importance of each goal (except 9 and 10) was significantly higher among females than males. In fact, males rated objective 9 (escape from everyday life) significantly higher than females.
- In RO, females rated the importance of goals 1–7 and 10 (managing stress) significantly higher than males, whereas goal 8 (pleasure from physical activity) was evaluated as less important by females.

### 3.4 Type of goal x country interaction at each level of gender

The pairwise multiple comparisons with Bonferroni correction indicated that among females, one of the largest differences occurred in the evaluation of the importance of objective 7 (boosting confidence, gaining appreciation from others) between PL and EG (*diff* = −1.35; *p* < 0.001), as well as PL and IR (*diff* = −1.31; *p* < 0.001). The Polish women assessed goal 7 as significantly less important (*M* = 3.04; SD = 1.49) than women in EG (*M* = 4.38; SD = 0.96) and IR (*M _=_* 4.35; SD *_=_* 1.03).

A relatively big difference was also shown regarding target 6 (being physically active and fit according to fashion) between females in IR and PL (*diff _=_* 1.37; *p* < 0.001), IR and IT (*diff _=_* 1.12; *p* < 0.001), as well as EG and PL (*diff _=_* 1.01; *p* < 0.001). The Polish women assessed goal 6 as significantly less important (*M _=_* 3.02; SD *_=_* 1.39) than women in IR (*M _=_* 4.39; SD *_=_* 0.89) and EG (*M _=_* 4.03; SD *_=_* 1.11). The women in IT also rated objective 6 as less important (*M _=_* 3.27; SD *_=_* 1.32) than women in IR.

The next biggest difference was observed in the ratings of objective 9 (escape from everyday life) between females in KZ and IT (*diff _=_* −1.20; *p* < 0.001), PL (*diff _=_* −1.01; *p* < 0.001), and RO (*diff _=_* −1.25; *p* < 0.001). It occurred that women in KZ considered this type of goal as significantly less important (*M _=_* 2.59; SD *_=_* 1.40) than women in IT (*M _=_* 3.78; SD *_=_* 1.24), PL (*M _=_* 3.59; SD *_=_* 1.16) and RO (*M _=_* 3.83; SD *_=_* 1.13). All PMCs were presented in Supplementary Table 1.

The PMCs with Bonferroni correction indicated that one of the largest differences occurred in the evaluation of the importance of objective 4 (Fit, shapely body) between males in RO and CN (*diff _=_* −1.11; *p* < 0.001), EG (*diff _=_* −1.12; p < 0.001), and IR (*diff _=_* −1.08; *p* < 0.001). The analysis showed that males in RO assessed goal 4 as significantly less important (*M _=_* 3.21; SD *_=_* 1.24) than males in EG (*M _=_* 4.33; SD *_=_* 0.81) and IR (*M _=_* 4.29; SD *_=_* 0.81).

The biggest difference was also shown in evaluating target 6 (being physically active and fit according to fashion) between males in PL and EG (*diff _=_* −1.77; *p* < 0.001), IR (*diff _=_* −1.73; *p* < 0.001), IT (*diff _=_* −1.33; *p* < 0.001), KZ (*diff _=_* −1.40; *p* < 0.001), as well as between males in RO and EG (*diff _=_* −1.18; *p* < 0.001) and IR (*diff _=_* −1.14; *p* < 0.001). The Polish men assessed goal 6 as significantly less important (*M _=_* 2.46; SD *_=_* 1.41) than men in EG (*M _=_* 4.22; SD *_=_* 0.94), IR (*M _=_* 4.19; SD *_=_* 1.00), and KZ (*M _=_* 3.86; SD *_=_* 1.26). Additionally, men in RO rated objective 6 as less important (*M _=_* 3.04; SD *_=_* 1.34) than men in EG and IR.

Another relatively relevant difference was observed in the ratings of objective 7 (boosting confidence, gaining appreciation from others) between males in PL and CN (*diff _=_* −1.23; *p* < 0.001), EG (*diff _=_* −1.61; *p* < 0.001), IR (*diff _=_* −1.26; *p* < 0.001), IT (*diff _=_* −1.12; *p* < 0.001), and KZ (*diff _=_* −1.22; *p* < 0.001). It turned out that men in PL considered this type of goal significantly less important (*M _=_* 2.74; SD *_=_* 1.32) than men in CN (*M _=_* 3.97; SD *_=_* 1.04), EG (*M _=_* 4.35; SD *_=_ 0.*84), IR (*M _=_* 4.00; SD *_=_* 1.06), IT (*M _=_* 3.86; SD *_=_* 1.15), and KZ (*M _=_* 3.95; SD *_=_* 1.24).

The next high difference among males in goal importance ratings between countries was observed with respect to goal 9 (escape from everyday life) between KZ and CN (*diff _=_* −1.00; *p* < 0.001), IT (*diff _=_* −1.13; *p* < 0.001), PL (*diff _=_* −1.13; *p* < 0.001), and RO (*diff _=_* −1.01; *p* < 0.001). The men in KZ rated this type of goal significantly lower (*M _=_* 2.67; SD *_=_* 1.44) than men in CN (*M _=_* 3.67; SD *_=_* 1.16), IT (*M _=_* 3.79; SD *_=_* 1.29), PL (*M _=_* 3.80; SD *_=_* 1.17), and RO (*M _=_* 3.67; SD *_=_* 1.11).

Additionally, there was a relevant difference in ratings of target 12 (promoting PA) between males in PL and IT (*diff _=_* −1.02; *p* < 0.001). The Polish men assessed this objective as less important (*M _=_* 3.24; SD *_=_* 1.42) than Italian men (*M _=_* 4.26; SD *_=_* 0.93) (Supplementary Table 1—only significant results at a level < 0.05 are presented due to the extensive data set).

## 4 Discussion

### 4.1 Main findings

Contemporary research indicates that the most significant factor threatening health is a sedentary lifestyle (Czarnecki et al., 2023). The World Health Organization has identified the promotion of physical activity as a major goal of modern public health strategies (Biernat, 2011). For the promotion of physical health to be effective, it is essential to understand the motivators for engaging in it. Therefore, two questions were posed in this study: Does gender differentiate the motives for physical activity in PL, CN, IR, KZ, EG, RO, and IT? Does nationality determine motivation for physical activity?

Considering the first research question, the results showed that there are several significant differences between women and men in different countries; however, the strength of the gender-country interaction effect was small. The results show that in Poland, Iran, Egypt, and Romania, women declared the importance of the goal defined as a “physical fitness and being in shape” higher than men. In addition, women from Kazakhstan, Poland, and Romania indicated a “health-related” goal as more important than men. In Iran, Poland and Romania, the motivation described as “wellbeing” is lower among men than women. Similar results were obtained by Lipowski and Ussorowska (2018), indicating that physical activity undertaken by women serves health and wellbeing. The results obtained can be substantiated by referring to gender-stereotypical demands, according to which physical beauty is required of women. Gender-stereotypical demands, particularly those related to physical beauty, place significant and disproportionate pressures on women, leading to both social and economic consequences. The existence of a “beauty premium” in the labor market highlights how women are often judged based on their appearance, which can both benefit and penalize them depending on how well they meet societal expectations (Andreoni and Petrie, 2008). This notion is reinforced by the prescriptive beauty norm (PBN), which compels women to continuously pursue beauty to maintain social and professional status. The PBN reflects and perpetuates gender hierarchies, particularly in the workplace, where women in high-power roles are subjected to stricter beauty standards than men (Ramati-Ziber et al., 2020). Media representations further amplify these demands by portraying idealized beauty standards, suggesting that adhering to specific beauty practices is essential for a woman’s attractiveness and social success (Emeksiz, 2021). These socio-psychological effects can lead to body dissatisfaction and the prioritization of physical appearance over other personal attributes, ultimately reinforcing the gender hierarchy and maintaining the social control of women based on their looks (Shyian et al., 2021).

Another observation from the current study was that women from Poland, Romania, and Iran differed from men in most of the 12 PA motives analyzed, with the exception of the motive: “escape from everyday life.” These gender differences highlight the influence of sociocultural factors on physical activity motivations, which vary based on gender and the country’s specific social and cultural context. For instance, studies on physical activity in Iran have shown significant gender-specific patterns, with men engaging more frequently in physical activities across various domains, while women showed reduced engagement, particularly in work-related physical activity (Koohpayehzadeh et al., 2014). Similarly, in Poland and Romania, traditional gender roles continue to shape women’s involvement in physical activities, with cultural expectations and health-related motivations playing a significant role in determining the extent and nature of their participation (Kuśnierz et al., 2020).

On the other hand, when observing the motives of males, they scored substantially higher on some specific motives, and those differences can be attributed to cultural, social, and psychological factors that shape gender roles and expectations. Italian and Egyptian males, who showed a higher motivation for “being physically active and fit according to fashion,” likely reflect societal pressures where physical appearance and fashionability are culturally significant, particularly among men who associate fitness with social status and attractiveness (Szabo et al., 2019). Italian males also rated the motive of “promoting physical activity by setting a behavioral example” higher than females, suggesting that men in this context might feel a stronger societal or cultural responsibility to be role models in physical activity, perhaps due to traditional gender roles that emphasize leadership and public behavior in men (Molanorouzi et al., 2015). In Kazakhstan, males scored higher on the motive of “enjoying the company of others” compared to females, which may indicate that social interaction is a stronger motivator for men in this region, possibly due to cultural norms that promote male bonding through group activities (Guérin, 2013). Polish males, who placed a higher importance on “escaping from everyday life,” might reflect a gender-specific coping mechanism where men use physical activity as an escape from stress or routine, which aligns with findings that men are more likely to use physical activity as a stress relief tool (Bednarek et al., 2016). Finally, Romanian males’ higher motivation for “finding pleasure in physical activity” compared to females suggests that men may derive more intrinsic enjoyment from physical activities, potentially due to a societal emphasis on male participation in sports and physical competition from a young age. These variations highlight how gendered cultural expectations and societal norms influence the motivation for physical activity differently across countries (Molanorouzi et al., 2015).

The results relating to the second research question showed that Polish women differ from women in other countries surveyed in two specific motives, with these motives also being lower among Polish men. Specifically, Polish women exhibited a lower level of the sixth PA motive, “engaging in physical activity and staying fit according to fashion,” compared to Iranian and Egyptian women. Similarly, Polish men scored significantly lower on this motive than men from Iran, Egypt, and Kazakhstan. The second motive, “boosting confidence and gaining appreciation from others,” was also lower in Polish women compared to Iranian and Egyptian women. Polish men similarly showed lower levels of this motive compared to men from Iran, Egypt, and Kazakhstan. Furthermore, Polish men differed from Italian men in having a lower level of the twelfth PA motive, “promoting physical activity by setting a behavioral example.” On the other hand, Italian women exhibited lower levels of the PA goal “engaging in physical activity and staying fit according to fashion” compared to Iranian women. Additionally, Kazakh women and men showed lower levels of the ninth PA motive, “escaping from everyday life,” compared to Polish, Italian, and Romanian women and men. Kazakh men also demonstrated lower levels of this motive compared to Chinese men. Romanian men, in turn, showed lower levels of the PA motives “engaging in physical activity and staying fit according to fashion” and “achieving a fit and shapely body” compared to Egyptian and Iranian men. Moreover, in the latter motive, Romanian men scored lower than Chinese men as well.

The cultural differences in physical activity motives, particularly in the comparison between Polish, Iranian, Egyptian, Kazakh, Chinese, and Romanian men and women, are well explained by several studies that highlight how cultural norms, values, and socio-economic conditions shape the motivations for engaging in physical activity. For instance, Mirsafian (2016) discusses the role of cultural values and societal norms in shaping physical activity participation, particularly noting how Iranian women’s participation is hindered by societal attitudes toward women in sports, while Polish students demonstrate more freedom in their physical activity engagement due to different cultural and socio-economic conditions. Similarly, Bednarek et al. (2016) compare Polish and Turkish students, showing that Polish students engage in more vigorous physical activity, which might be influenced by different educational and health promotion policies in Poland compared to Turkey. Additionally, research on Polish and Chinese sociocultural attitudes by Guo et al. (2023) highlights how cultural adaptation goals impact motivations for physical activity, particularly how young men and women in China and Poland differ in their focus on physical appearance and social motivations in relation to sports. These studies demonstrate how variations in cultural values, economic conditions, and gender norms play significant roles in influencing the motives behind physical activity across different countries.

### 4.2 Practical implications

The results of the study, which show differences in the motives for physical activity in different countries and between genders, have a number of practical implications for health policy, education, marketing, and the promotion of physical fitness. Firstly, the cultural differences revealed indicate the need to design campaigns promoting physical activity in a way that is tailored to local values and priorities. This means that in countries where health motives dominate, programs should place particular emphasis on disease prevention and mental and physical wellbeing, while in countries where competition or physical fitness are key, messages can be based on narratives of achievement and challenge. In physical education, it is worth adapting curricula to the dominant motives in a given group in order to strengthen student engagement by aligning content with their expectations. For example, physical activities combining health and esthetic elements (such as dance or Pilates) may be more effective for girls, while activities involving competition (such as team sports) may be more effective for boys. Allowing students to choose their activities will contribute to a greater sense of autonomy. Marketing activities should take into account the language and content of the message directed at the audience. In addition, it is worth using ambassadors and opinion leaders whose lifestyle is consistent with the dominant themes in a given culture to promote physical fitness. Sports infrastructure planning should also take into account preferences resulting from prevailing goals—e.g., greater emphasis on recreational facilities where relaxation is a priority or sports fields and training halls in competitive contexts. It is also important that coaches and instructors are trained to recognize participants’ motivational profiles, which will allow them to individualize their approach. It is also worth developing applications and digital tools that personalize training goals and motivational messages based on the user’s cultural profile.

### 4.3 Limitations

The current study has some limitations. One of the primary limitations of this study is the reliance on self-reported data for assessing physical activity motives across different countries. Self-reported measures are susceptible to bias, including social desirability bias, which could lead participants to overreport or underreport their true motivations. Furthermore, the study did not account for potential confounding variables such as socio-economic status, education, or access to sports facilities (some of these variables were only described), all of which can significantly impact an individual’s motivation for physical activity. The study was correlational in nature, so it is impossible to draw causal conclusions.

### 4.4 Further research directions

Future research should focus on using more objective measures of physical activity and motivation, such as accelerometers or ecological momentary assessments, to complement self-reported data. Expanding the study to include a broader range of countries and cultural contexts could provide more comprehensive insights into the global diversity of physical activity motivations. Longitudinal studies could also be valuable to examine how motivations for physical activity evolve over time, particularly in response to changing cultural norms or public health interventions. Goals are highly malleable structures that can be modified over time (Brazo-Sayavera et al., 2021). Moreover, future studies should explore the impact of additional factors such as socio-economic status, education, age, and access to resources on physical activity motives. Incorporating qualitative methods, such as interviews or focus groups, could offer deeper insights into the cultural and gender-specific nuances that shape physical activity motivations. Lastly, considering interventions that target specific cultural and gender-related barriers to physical activity could help develop more tailored public health strategies to promote active lifestyles globally.

## 5 Conclusion

This study provides valuable insights into the gender and cross-cultural differences in physical activity motives across several countries, including Poland, China, Iran, Kazakhstan, Egypt, Romania, and Italy. The findings suggest that women, particularly in Poland, Iran, and Romania, are more motivated by health-related goals and wellbeing, likely influenced by societal pressures around physical appearance. On the other hand, men’s motivations were more varied, with influences from cultural and social norms, such as enjoying social interactions or using physical activity as an escape from daily stress. These findings emphasize the importance of considering cultural and gender-specific factors when developing public health strategies aimed at promoting physical activity. Addressing these nuanced motivations could enhance the effectiveness of interventions designed to increase physical activity across diverse populations.

The research underscores the potential of interdisciplinary collaboration between psychology, public health, and sociology in shaping sustainable behavioral interventions.

## Data Availability

The raw data supporting the conclusions of this article will be made available by the authors, without undue reservation.
