# Supplementary material for: The motivational function of goals in physical activity: a cross-cultural comparison
Source: Front Psychol. 2026 Jan 6;16:1642206. doi: 10.3389/fpsyg.2025.1642206 (PMC12815830; doi:10.3389/fpsyg.2025.1642206)
Supplement: Supplementary file 1 [file Table_1.docx]

**Supplementary Table** **1.** *The results of multiple comparisons significance of differences in importance of the goal between countries depending on the type of target among males and females*

| Gender | Goal | Country (I) | (J) | Difference in means (I-J) | *SE* | *p_bonf_* | Gender | Goal | Country (I) | (J) | Difference in means (I-J) | *SE* | *p_bonf_* |
| --- | --- | --- | --- | --- | --- | --- | --- | --- | --- | --- | --- | --- | --- |
|  |  |  |  |  |  |  |  |  |  |  |  |  |  |
| Female | 1 | EG | PL | -0.26^**^ | 0.07 | .002 | Male | 1 | CN | KZ | 0.21^*^ | 0.07 | .044 |
|  |  | PL | EG | 0.26^**^ | 0.07 | .002 |  |  |  | PL | 0.30^***^ | 0.07 | <.001 |
|  | 2 | CN | IR | -0.40^***^ | 0.07 | <.001 |  |  |  | RO | 0.33^**^ | 0.09 | .007 |
|  |  |  | IT | -0.24^*^ | 0.07 | .015 |  |  | IR | KZ | 0.26^*^ | 0.08 | .018 |
|  |  |  | PL | -0.33^***^ | 0.06 | <.001 |  |  |  | PL | 0.35^***^ | 0.08 | <.001 |
|  |  | EG | IR | -0.42^***^ | 0.08 | <.001 |  |  |  | RO | 0.38^**^ | 0.10 | .003 |
|  |  |  | IT | -0.26^*^ | 0.08 | .035 |  |  | IT | KZ | 0.28^*^ | 0.08 | .019 |
|  |  |  | PL | -0.35^***^ | 0.07 | <.001 |  |  |  | PL | 0.37^***^ | 0.08 | <.001 |
|  |  | IR | CN | 0.40^***^ | 0.07 | <.001 |  |  |  | RO | 0.40^**^ | 0.11 | .003 |
|  |  |  | EG | 0.42^***^ | 0.08 | <.001 |  |  | KZ | CN | -0.21^*^ | 0.07 | .044 |
|  |  |  | KZ | 0.32^**^ | 0.09 | .008 |  |  |  | IR | -0.26^*^ | 0.08 | .018 |
|  |  |  | RO | 0.31^**^ | 0.09 | .008 |  |  |  | IT | -0.28^*^ | 0.08 | .019 |
|  |  | IT | CN | 0.24^*^ | 0.07 | .015 |  |  | PL | CN | -0.30^***^ | 0.07 | <.001 |
|  |  |  | EG | 0.26^*^ | 0.08 | .035 |  |  |  | IR | -0.35^***^ | 0.08 | <.001 |
|  |  | KZ | IR | -0.32^**^ | 0.09 | .008 |  |  |  | IT | -0.37^***^ | 0.08 | <.001 |
|  |  |  | PL | -0.25^*^ | 0.08 | .034 |  |  | RO | CN | -0.33^**^ | 0.09 | .007 |
|  |  | PL | CN | 0.33^***^ | 0.06 | <.001 |  |  |  | IR | -0.38^**^ | 0.10 | .003 |
|  |  |  | EG | 0.35^***^ | 0.07 | <.001 |  |  |  | IT | -0.40^**^ | 0.11 | .003 |
|  |  |  | KZ | 0.25^*^ | 0.08 | .034 |  | 2 | CN | EG | 0.33^***^ | 0.07 | <.001 |
|  |  |  | RO | 0.24^*^ | 0.08 | .036 |  |  | EG | CN | -0.33^***^ | 0.07 | <.001 |
|  |  | RO | IR | -0.31^**^ | 0.09 | .008 |  |  |  | IR | -0.29^**^ | 0.08 | .003 |
|  |  |  | PL | -0.24^*^ | 0.08 | .036 |  |  |  | IT | -0.51^***^ | 0.08 | <.001 |
|  | 3 | CN | IR | 0.30^*^ | 0.09 | .026 |  |  |  | KZ | -0.36^***^ | 0.07 | <.001 |
|  |  |  | KZ | 0.53^***^ | 0.10 | <.001 |  |  |  | PL | -0.40^***^ | 0.07 | <.001 |
|  |  |  | RO | 0.38^***^ | 0.09 | .001 |  |  | IR | EG | 0.29^**^ | 0.08 | .003 |
|  |  | EG | KZ | 0.39^*^ | 0.11 | .010 |  |  | IT | EG | 0.51^***^ | 0.08 | <.001 |
|  |  | IR | CN | -0.30^*^ | 0.09 | .026 |  |  |  | RO | 0.38^*^ | 0.11 | .010 |
|  |  | KZ | CN | -0.53^***^ | 0.10 | <.001 |  |  | KZ | EG | 0.36^***^ | 0.07 | <.001 |
|  |  |  | EG | -0.39^*^ | 0.11 | .010 |  |  | PL | EG | 0.40^***^ | 0.07 | <.001 |
|  |  |  | PL | -0.32^*^ | 0.10 | .030 |  |  | RO | IT | -0.38^*^ | 0.11 | .010 |
|  |  | PL | KZ | 0.32^*^ | 0.10 | .030 |  | 3 | EG | PL | 0.35^**^ | 0.09 | .002 |
|  |  | RO | CN | -0.38^**^ | 0.09 | .001 |  |  |  | RO | 0.56^***^ | 0.12 | <.001 |
|  | 4 | CN | IR | -0.28^**^ | 0.07 | .003 |  |  | IT | PL | 0.36^*^ | 0.11 | .019 |
|  |  |  | IT | 0.31^***^ | 0.07 | <.001 |  |  |  | RO | 0.57^**^ | 0.14 | .001 |
|  |  |  | RO | 0.37^***^ | 0.08 | <.001 |  |  | KZ | RO | 0.48^**^ | 0.13 | .004 |
|  |  | EG | IR | -0.33^**^ | 0.09 | .002 |  |  | PL | EG | -0.35^**^ | 0.09 | .002 |
|  |  |  | IT | 0.27^*^ | 0.08 | .025 |  |  |  | IT | -0.36^*^ | 0.11 | .019 |
|  |  |  | RO | 0.33^**^ | 0.09 | .003 |  |  | RO | EG | -0.56^***^ | 0.12 | <.001 |
|  |  | IR | CN | 0.28^**^ | 0.07 | .003 |  |  |  | IT | -0.57^**^ | 0.14 | .001 |
|  |  |  | EG | 0.33^**^ | 0.09 | .002 |  |  |  | KZ | -0.48^**^ | 0.13 | .004 |
|  |  |  | IT | 0.60^***^ | 0.09 | <.001 |  | 4 | CN | IT | 0.39^***^ | 0.08 | <.001 |
|  |  |  | PL | 0.37^***^ | 0.08 | <.001 |  |  |  | PL | 0.48^***^ | 0.07 | <.001 |
|  |  |  | RO | 0.65^***^ | 0.09 | <.001 |  |  |  | RO | 1.11^***^ | 0.10 | <.001 |
|  |  | IT | CN | -0.31^***^ | 0.07 | <.001 |  |  | EG | IT | 0.40^***^ | 0.09 | <.001 |
|  |  |  | EG | -0.27^*^ | 0.08 | .025 |  |  |  | PL | 0.49^***^ | 0.07 | <.001 |
|  |  |  | IR | -0.60^***^ | 0.09 | <.001 |  |  |  | RO | 1.12^***^ | 0.10 | <.001 |
|  |  |  | KZ | -0.34^**^ | 0.09 | .003 |  |  | IR | IT | 0.35^**^ | 0.09 | .003 |
|  |  |  | PL | -0.23^*^ | 0.07 | .034 |  |  |  | PL | 0.45^***^ | 0.08 | <.001 |
|  |  | KZ | IT | 0.34^**^ | 0.09 | .003 |  |  |  | RO | 1.08^***^ | 0.11 | <.001 |
|  |  |  | RO | 0.40^***^ | 0.09 | <.001 |  |  | IT | CN | -0.39^***^ | 0.08 | <.001 |
|  |  | PL | IR | -0.37^***^ | 0.08 | <.001 |  |  |  | EG | -0.40^***^ | 0.09 | <.001 |
|  |  |  | IT | 0.23^*^ | 0.07 | .034 |  |  |  | IR | -0.35^**^ | 0.09 | .003 |
|  |  |  | RO | 0.29^**^ | 0.08 | .004 |  |  |  | RO | 0.73^***^ | 0.11 | <.001 |
|  |  | RO | CN | -0.37^***^ | 0.08 | <.001 |  |  | KZ | PL | 0.30^**^ | 0.08 | .002 |
|  |  |  | EG | -0.33^**^ | 0.09 | .003 |  |  |  | RO | 0.93^***^ | 0.10 | <.001 |
|  |  |  | IR | -0.65^***^ | 0.09 | <.001 |  |  | PL | CN | -0.48^***^ | 0.07 | <.001 |
|  |  |  | KZ | -0.40^***^ | 0.09 | <.001 |  |  |  | EG | -0.49^***^ | 0.07 | <.001 |
|  |  |  | PL | -0.29^**^ | 0.08 | .004 |  |  |  | IR | -0.45^***^ | 0.08 | <.001 |
|  | 5 | CN | IR | -0.34^***^ | 0.06 | <.001 |  |  |  | KZ | -0.30^**^ | 0.08 | .002 |
|  |  |  | IT | -0.35^***^ | 0.06 | <.001 |  |  |  | RO | 0.63^***^ | 0.10 | <.001 |
|  |  |  | PL | -0.28^***^ | 0.05 | <.001 |  |  | RO | CN | -1.11^***^ | 0.10 | <.001 |
|  |  | EG | IR | -0.52^***^ | 0.07 | <.001 |  |  |  | EG | -1.12^***^ | 0.10 | <.001 |
|  |  |  | IT | -0.52^***^ | 0.07 | <.001 |  |  |  | IR | -1.08^***^ | 0.11 | <.001 |
|  |  |  | KZ | -0.31^**^ | 0.07 | .001 |  |  |  | IT | -0.73^***^ | 0.11 | <.001 |
|  |  |  | PL | -0.45^***^ | 0.06 | <.001 |  |  |  | KZ | -0.93^***^ | 0.10 | <.001 |
|  |  |  | RO | -0.26^**^ | 0.07 | .007 |  |  |  | PL | -0.63^***^ | 0.10 | <.001 |
|  |  | IR | CN | 0.34^***^ | 0.06 | <.001 |  | 5 | CN | EG | 0.32^***^ | 0.06 | <.001 |
|  |  |  | EG | 0.52^***^ | 0.07 | <.001 |  |  |  | IT | -0.27^**^ | 0.07 | .002 |
|  |  |  | RO | 0.26^**^ | 0.07 | .008 |  |  | EG | CN | -0.32^***^ | 0.06 | <.001 |
|  |  | IT | CN | 0.35^***^ | 0.06 | <.001 |  |  |  | IR | -0.40^***^ | 0.06 | <.001 |
|  |  |  | EG | 0.52^***^ | 0.07 | <.001 |  |  |  | IT | -0.58^***^ | 0.07 | <.001 |
|  |  |  | RO | 0.27^**^ | 0.07 | .005 |  |  |  | KZ | -0.27^***^ | 0.06 | <.001 |
|  |  | KZ | EG | 0.31^**^ | 0.07 | .001 |  |  |  | PL | -0.28^***^ | 0.06 | <.001 |
|  |  | PL | CN | 0.28^***^ | 0.05 | <.001 |  |  | IR | EG | 0.40^***^ | 0.06 | <.001 |
|  |  |  | EG | 0.45^***^ | 0.06 | <.001 |  |  | IT | CN | 0.27^**^ | 0.07 | .002 |
|  |  |  | RO | 0.20^*^ | 0.06 | .042 |  |  |  | EG | 0.58^***^ | 0.07 | <.001 |
|  |  | RO | EG | 0.26^**^ | 0.07 | .007 |  |  |  | KZ | 0.32^***^ | 0.07 | <.001 |
|  |  |  | IR | -0.26^**^ | 0.07 | .008 |  |  |  | PL | 0.31^***^ | 0.07 | <.001 |
|  |  |  | IT | -0.27^**^ | 0.07 | .005 |  |  |  | RO | 0.44^***^ | 0.09 | <.001 |
|  |  |  | PL | -0.20^*^ | 0.06 | .042 |  |  | KZ | EG | 0.27^***^ | 0.06 | <.001 |
|  | 6 | CN | EG | -0.57^***^ | 0.09 | <.001 |  |  |  | IT | -0.32^***^ | 0.07 | <.001 |
|  |  |  | IR | -0.93^***^ | 0.10 | <.001 |  |  | PL | EG | 0.28^***^ | 0.06 | <.001 |
|  |  |  | PL | 0.44^***^ | 0.08 | <.001 |  |  |  | IT | -0.31^***^ | 0.07 | <.001 |
|  |  | EG | CN | 0.57^***^ | 0.09 | <.001 |  |  | RO | IT | -0.44^***^ | 0.09 | <.001 |
|  |  |  | IR | -0.36^*^ | 0.11 | .026 |  | 6 | CN | EG | -0.79^***^ | 0.09 | <.001 |
|  |  |  | IT | 0.76^***^ | 0.11 | <.001 |  |  |  | IR | -0.76^***^ | 0.10 | <.001 |
|  |  |  | PL | 1.01^***^ | 0.09 | <.001 |  |  |  | IT | -0.35^*^ | 0.11 | .030 |
|  |  |  | RO | 0.59^***^ | 0.11 | <.001 |  |  |  | KZ | -0.43^***^ | 0.10 | <.001 |
|  |  | IR | CN | 0.93^***^ | 0.10 | <.001 |  |  |  | PL | 0.97^***^ | 0.09 | <.001 |
|  |  |  | EG | 0.36^*^ | 0.11 | .026 |  |  | EG | CN | 0.79^***^ | 0.09 | <.001 |
|  |  |  | IT | 1.12^***^ | 0.11 | <.001 |  |  |  | IT | 0.44^**^ | 0.11 | .002 |
|  |  |  | KZ | 0.66^***^ | 0.12 | <.001 |  |  |  | KZ | 0.37^**^ | 0.10 | .003 |
|  |  |  | PL | 1.37^***^ | .10 | <.001 |  |  |  | PL | 1.77^***^ | 0.09 | <.001 |
|  |  |  | RO | 0.95^***^ | 0.12 | <.001 |  |  |  | RO | 1.18^***^ | 0.13 | <.001 |
|  |  | IT | EG | -0.76^***^ | 0.11 | <.001 |  |  | IR | CN | 0.76^***^ | 0.10 | <.001 |
|  |  |  | IR | -1.12^***^ | .11 | <.001 |  |  |  | IT | 0.40^*^ | 0.12 | .020 |
|  |  |  | KZ | -0.46^**^ | 0.12 | .002 |  |  |  | PL | 1.73^***^ | 0.11 | <.001 |
|  |  | KZ | IR | -0.66^***^ | 0.12 | <.001 |  |  |  | RO | 1.14^***^ | 0.14 | <.001 |
|  |  |  | IT | 0.46^**^ | 0.12 | .002 |  |  | IT | CN | 0.35^*^ | 0.11 | .030 |
|  |  |  | PL | 0.71^***^ | 0.11 | <.001 |  |  |  | EG | -0.44^**^ | 0.11 | .002 |
|  |  | PL | CN | -0.44^***^ | 0.08 | <.001 |  |  |  | IR | -0.40^*^ | 0.12 | .020 |
|  |  |  | EG | -1.01^***^ | 0.09 | <.001 |  |  |  | PL | 1.33^***^ | 0.12 | <.001 |
|  |  |  | IR | -1.37^***^ | 0.10 | <.001 |  |  |  | RO | 0.74^***^ | 0.15 | <.001 |
|  |  |  | KZ | -0.71^***^ | 0.11 | <.001 |  |  | KZ | CN | 0.43^***^ | 0.10 | <.001 |
|  |  |  | RO | -0.42^**^ | 0.10 | .001 |  |  |  | EG | -0.37^**^ | 0.10 | .003 |
|  |  | RO | EG | -0.59^***^ | 0.11 | <.001 |  |  |  | PL | 1.4^***^ | 0.10 | <.001 |
|  |  |  | IR | -0.95^***^ | 0.12 | <.001 |  |  |  | RO | 0.82^***^ | 0.14 | <.001 |
|  |  |  | PL | 0.42^**^ | 0.10 | .001 |  |  | PL | CN | -0.97^***^ | 0.09 | <.001 |
|  | 7 | CN | EG | -0.39^***^ | 0.09 | <.001 |  |  |  | EG | -1.77^***^ | 0.09 | <.001 |
|  |  |  | IR | -0.35^**^ | 0.09 | .002 |  |  |  | IR | -1.73^***^ | 0.11 | <.001 |
|  |  |  | IT | 0.31^**^ | 0.09 | .007 |  |  |  | IT | -1.33^***^ | 0.12 | <.001 |
|  |  |  | PL | 0.96^***^ | 0.07 | <.001 |  |  |  | KZ | -1.4^***^ | 0.10 | <.001 |
|  |  | EG | CN | 0.39^***^ | 0.09 | <.001 |  |  |  | RO | -0.59^***^ | 0.13 | <.001 |
|  |  |  | IT | 0.70^***^ | 0.10 | <.001 |  |  | RO | EG | -1.18^***^ | 0.13 | <.001 |
|  |  |  | KZ | 0.40^**^ | 0.11 | .005 |  |  |  | IR | -1.14^***^ | 0.14 | <.001 |
|  |  |  | PL | 1.35^***^ | 0.09 | <.001 |  |  |  | IT | -0.74^***^ | 0.15 | <.001 |
|  |  |  | RO | 0.60^***^ | 0.11 | <.001 |  |  |  | KZ | -0.82^***^ | 0.14 | <.001 |
|  |  | IR | CN | 0.35^**^ | 0.09 | .002 |  |  |  | PL | 0.59^***^ | 0.13 | <.001 |
|  |  |  | IT | 0.67^***^ | 0.11 | <.001 |  | 7 | CN | EG | -0.38^***^ | 0.08 | <.001 |
|  |  |  | KZ | 0.37^*^ | 0.11 | .020 |  |  |  | PL | 1.23^***^ | 0.09 | <.001 |
|  |  |  | PL | 1.31^***^ | 0.09 | <.001 |  |  |  | RO | 0.56^***^ | 0.12 | <.001 |
|  |  |  | RO | 0.57^***^ | 0.11 | <.001 |  |  | EG | CN | 0.38^***^ | 0.08 | <.001 |
|  |  | IT | CN | -0.31^**^ | 0.09 | .007 |  |  |  | IR | 0.35^**^ | 0.10 | .006 |
|  |  |  | EG | -0.70^***^ | 0.10 | <.001 |  |  |  | IT | 0.49^***^ | 0.10 | <.001 |
|  |  |  | IR | -0.67^***^ | 0.11 | <.001 |  |  |  | KZ | 0.40^***^ | 0.09 | <.001 |
|  |  |  | PL | 0.65^***^ | 0.09 | <.001 |  |  |  | PL | 1.61^***^ | 0.09 | <.001 |
|  |  | KZ | EG | -0.40^**^ | 0.11 | .005 |  |  |  | RO | 0.94^***^ | 0.12 | <.001 |
|  |  |  | IR | -0.37^*^ | 0.11 | .020 |  |  | IR | EG | -0.35^**^ | 0.10 | .006 |
|  |  |  | PL | 0.94^***^ | 0.10 | <.001 |  |  |  | PL | 1.26^***^ | 0.10 | <.001 |
|  |  | PL | CN | -0.96^***^ | 0.07 | <.001 |  |  |  | RO | 0.59^***^ | 0.13 | <.001 |
|  |  |  | EG | -1.35^***^ | 0.09 | <.001 |  |  | IT | EG | -0.49^***^ | 0.10 | <.001 |
|  |  |  | IR | -1.31^***^ | 0.09 | <.001 |  |  |  | PL | 1.12^***^ | 0.11 | <.001 |
|  |  |  | IT | -0.65^***^ | 0.09 | <.001 |  |  |  | RO | 0.45^*^ | 0.14 | .023 |
|  |  |  | KZ | -0.94^***^ | 0.10 | <.001 |  |  | KZ | EG | -0.40^***^ | 0.09 | <.001 |
|  |  |  | RO | -0.74^***^ | 0.09 | <.001 |  |  |  | PL | 1.22^***^ | 0.09 | <.001 |
|  |  | RO | EG | -0.60^***^ | 0.11 | <.001 |  |  |  | RO | 0.54^***^ | 0.13 | <.001 |
|  |  |  | IR | -0.57^***^ | 0.11 | <.001 |  |  | PL | CN | -1.23^***^ | 0.09 | <.001 |
|  |  |  | PL | 0.74^***^ | 0.09 | <.001 |  |  |  | EG | -1.61^***^ | 0.09 | <.001 |
|  | 8 | CN | RO | 0.49^***^ | 0.07 | <.001 |  |  |  | IR | -1.26^***^ | 0.10 | <.001 |
|  |  | EG | IR | -0.31^**^ | 0.08 | .003 |  |  |  | IT | -1.12^***^ | 0.11 | <.001 |
|  |  |  | IT | -0.25^*^ | 0.08 | .038 |  |  |  | KZ | -1.22^***^ | 0.09 | <.001 |
|  |  |  | PL | -0.22^*^ | 0.07 | .024 |  |  |  | RO | -0.67^***^ | 0.13 | <.001 |
|  |  |  | RO | 0.32^**^ | 0.08 | .002 |  |  | RO | CN | -0.56^***^ | 0.12 | <.001 |
|  |  | IR | EG | 0.31^**^ | 0.08 | .003 |  |  |  | EG | -0.94^***^ | 0.12 | <.001 |
|  |  |  | KZ | 0.28^*^ | 0.09 | .033 |  |  |  | IR | -0.59^***^ | 0.13 | <.001 |
|  |  |  | RO | 0.63^***^ | 0.09 | <.001 |  |  |  | IT | -0.45^*^ | 0.14 | .023 |
|  |  | IT | EG | 0.25^*^ | 0.08 | .038 |  |  |  | KZ | -0.54^***^ | 0.13 | <.001 |
|  |  |  | RO | 0.57^***^ | 0.08 | <.001 |  |  |  | PL | 0.67^***^ | 0.13 | <.001 |
|  |  | KZ | IR | -0.28^*^ | 0.09 | .033 |  | 8 | EG | IT | -0.34^***^ | 0.08 | <.001 |
|  |  |  | RO | 0.35^**^ | 0.09 | .002 |  |  | IT | EG | 0.34^***^ | 0.08 | <.001 |
|  |  | PL | EG | 0.22^*^ | 0.07 | .024 |  |  |  | RO | 0.38^**^ | 0.11 | .007 |
|  |  |  | RO | 0.55^***^ | 0.07 | <.001 |  |  | RO | IT | -0.38^**^ | 0.11 | .007 |
|  |  | RO | CN | -0.49^***^ | 0.07 | <.001 |  | 9 | CN | EG | 0.29^*^ | 0.09 | .023 |
|  |  |  | EG | -0.32^**^ | 0.08 | .002 |  |  |  | IR | 0.43^***^ | 0.10 | <.001 |
|  |  |  | IR | -0.63^***^ | 0.09 | <.001 |  |  |  | KZ | 1.00^***^ | 0.10 | <.001 |
|  |  |  | IT | -0.57^***^ | 0.08 | <.001 |  |  | EG | CN | -0.29^*^ | 0.09 | .023 |
|  |  |  | KZ | -0.35^**^ | 0.09 | .002 |  |  |  | IT | -0.41^**^ | 0.11 | .005 |
|  |  |  | PL | -0.55^***^ | 0.07 | <.001 |  |  |  | KZ | 0.72^***^ | 0.10 | <.001 |
|  | 9 | CN | KZ | 1.00^***^ | 0.10 | <.001 |  |  |  | PL | -0.42^***^ | 0.09 | <.001 |
|  |  | EG | KZ | 0.95^***^ | 0.12 | <.001 |  |  | IR | CN | -0.43^***^ | 0.10 | <.001 |
|  |  | IR | IT | -0.47^**^ | 0.11 | .001 |  |  |  | IT | -0.56^***^ | 0.12 | <.001 |
|  |  |  | KZ | 0.73^***^ | 0.12 | <.001 |  |  |  | KZ | 0.57^***^ | 0.11 | <.001 |
|  |  |  | RO | -0.52^***^ | 0.12 | <.001 |  |  |  | PL | -0.56^***^ | 0.11 | <.001 |
|  |  | IT | IR | 0.47^**^ | 0.11 | .001 |  |  |  | RO | -0.44^*^ | 0.14 | .041 |
|  |  |  | KZ | 1.20^***^ | 0.12 | <.001 |  |  | IT | EG | 0.41^**^ | 0.11 | .005 |
|  |  | KZ | CN | -1.00^***^ | 0.10 | <.001 |  |  |  | IR | 0.56^***^ | 0.12 | <.001 |
|  |  |  | EG | -0.95^***^ | 0.12 | <.001 |  |  |  | KZ | 1.13^***^ | 0.12 | <.001 |
|  |  |  | IR | -0.73^***^ | 0.12 | <.001 |  |  | KZ | CN | -1.00^***^ | 0.10 | <.001 |
|  |  |  | IT | -1.20^***^ | 0.12 | <.001 |  |  |  | EG | -0.72^***^ | 0.10 | <.001 |
|  |  |  | PL | -1.01^***^ | 0.11 | <.001 |  |  |  | IR | -0.57^***^ | 0.11 | <.001 |
|  |  |  | RO | -1.25^***^ | 0.12 | <.001 |  |  |  | IT | -1.13^***^ | 0.12 | <.001 |
|  |  | PL | KZ | 1.01^***^ | 0.11 | <.001 |  |  |  | PL | -1.13^***^ | 0.10 | <.001 |
|  |  | RO | IR | 0.52^***^ | 0.12 | <.001 |  |  |  | RO | -1.01^***^ | 0.14 | <.001 |
|  |  |  | KZ | 1.25^***^ | 0.12 | <.001 |  |  | PL | EG | 0.42^***^ | 0.09 | <.001 |
|  | 10 | CN | IR | -0.27^*^ | 0.08 | .021 |  |  |  | IR | 0.56^***^ | 0.11 | <.001 |
|  |  | EG | IR | -0.34^**^ | 0.09 | .005 |  |  |  | KZ | 1.13^***^ | 0.10 | <.001 |
|  |  | IR | CN | 0.27^*^ | 0.08 | .021 |  |  | RO | IR | 0.44^*^ | 0.14 | .041 |
|  |  |  | EG | 0.34^**^ | 0.09 | .005 |  |  |  | KZ | 1.01^***^ | 0.14 | <.001 |
|  |  |  | PL | 0.33^**^ | 0.08 | .001 |  | 10 | CN | EG | 0.26^**^ | 0.07 | .009 |
|  |  | PL | IR | -0.33^**^ | 0.08 | .001 |  |  |  | PL | 0.34^***^ | 0.08 | <.001 |
|  | 11 | CN | IR | -0.24^*^ | 0.07 | .022 |  |  |  | RO | 0.37^*^ | 0.11 | .014 |
|  |  |  | KZ | -0.25^*^ | 0.08 | .035 |  |  | EG | CN | -0.26^**^ | 0.07 | .009 |
|  |  | IR | CN | 0.24^*^ | 0.07 | .022 |  |  | KZ | PL | 0.32^**^ | 0.08 | .002 |
|  |  |  | RO | 0.34^**^ | 0.09 | .003 |  |  |  | RO | 0.35^*^ | 0.11 | .040 |
|  |  | IT | RO | 0.27^*^ | 0.09 | .039 |  |  | PL | CN | -0.34^***^ | 0.08 | <.001 |
|  |  | KZ | CN | 0.25^*^ | 0.08 | .035 |  |  |  | KZ | -0.32^**^ | 0.08 | .002 |
|  |  |  | RO | 0.35^**^ | 0.09 | .004 |  |  | RO | CN | -0.37^*^ | 0.11 | .014 |
|  |  | RO | IR | -0.34^**^ | 0.09 | .003 |  |  |  | KZ | -0.35^*^ | 0.11 | .040 |
|  |  |  | IT | -0.27^*^ | 0.09 | .039 |  | 11 | EG | KZ | -0.23^*^ | 0.07 | .042 |
|  |  |  | KZ | -0.35 | 0.09 | .004 |  |  | IT | PL | 0.28^*^ | 0.09 | .026 |
|  | 12 | CN | EG | -0.37^***^ | 0.08 | <.001 |  |  |  | RO | 0.36^*^ | 0.11 | .032 |
|  |  |  | IR | -0.49^***^ | 0.09 | <.001 |  |  | KZ | EG | 0.23^*^ | 0.07 | .042 |
|  |  |  | PL | 0.23^*^ | 0.07 | .016 |  |  |  | PL | 0.29^**^ | 0.08 | .003 |
|  |  | EG | CN | 0.37^***^ | 0.08 | <.001 |  |  |  | RO | 0.36^*^ | 0.10 | .010 |
|  |  |  | PL | 0.60^***^ | 0.09 | <.001 |  |  | PL | IT | -0.28^*^ | 0.09 | .026 |
|  |  |  | RO | 0.62^***^ | 0.10 | <.001 |  |  |  | KZ | -0.29^**^ | 0.08 | .003 |
|  |  | IR | CN | 0.49^***^ | 0.09 | <.001 |  |  | RO | IT | -0.36^*^ | 0.11 | .032 |
|  |  |  | IT | 0.37^**^ | 0.10 | .007 |  |  |  | KZ | -0.36^*^ | 0.10 | .010 |
|  |  |  | KZ | 0.35^*^ | 0.11 | .027 |  | 12 | CN | EG | -0.44^***^ | 0.08 | <.001 |
|  |  |  | PL | 0.72^***^ | 0.09 | <.001 |  |  |  | IR | -0.39^**^ | 0.09 | .001 |
|  |  |  | RO | 0.74^***^ | 0.11 | <.001 |  |  |  | IT | -0.51^***^ | 0.10 | <.001 |
|  |  | IT | IR | -0.37^**^ | 0.10 | .007 |  |  |  | KZ | -0.27^*^ | 0.09 | .042 |
|  |  |  | PL | 0.36^**^ | 0.09 | .001 |  |  |  | PL | 0.51^***^ | 0.08 | <.001 |
|  |  |  | RO | 0.38^**^ | 0.10 | .006 |  |  |  | RO | 0.39^*^ | 0.12 | .020 |
|  |  | KZ | IR | -0.35^*^ | 0.11 | .027 |  |  | EG | CN | 0.44^***^ | 0.08 | <.001 |
|  |  |  | PL | 0.37^**^ | 0.10 | .002 |  |  |  | PL | 0.95^***^ | 0.08 | <.001 |
|  |  |  | RO | 0.39^**^ | 0.11 | .008 |  |  |  | RO | 0.83^***^ | 0.12 | <.001 |
|  |  | PL | CN | -0.23^*^ | 0.07 | .016 |  |  | IR | CN | 0.39^**^ | 0.09 | .001 |
|  |  |  | EG | -0.60^***^ | 0.09 | <.001 |  |  |  | PL | 0.90^***^ | 0.10 | <.001 |
|  |  |  | IR | -0.72^***^ | 0.09 | <.001 |  |  |  | RO | 0.77^***^ | 0.13 | <.001 |
|  |  |  | IT | -0.36^**^ | 0.09 | .001 |  |  | IT | CN | 0.51^***^ | 0.10 | <.001 |
|  |  |  | KZ | -0.37^**^ | 0.10 | .002 |  |  |  | PL | 1.02^***^ | 0.10 | <.001 |
|  |  | RO | EG | -0.62^***^ | 0.10 | <.001 |  |  |  | RO | 0.90^***^ | 0.13 | <.001 |
|  |  |  | IR | -0.74^***^ | 0.11 | <.001 |  |  | KZ | CN | 0.27^*^ | 0.09 | .042 |
|  |  |  | IT | -0.38^**^ | 0.10 | .006 |  |  |  | PL | 0.78^***^ | 0.09 | <.001 |
|  |  |  | KZ | -0.39^**^ | 0.11 | .008 |  |  |  | RO | 0.65^***^ | 0.12 | <.001 |
|  |  |  |  |  |  |  |  |  | PL | CN | -0.51^***^ | 0.08 | <.001 |
|  |  |  |  |  |  |  |  |  |  | EG | -0.95^***^ | 0.08 | <.001 |
|  |  |  |  |  |  |  |  |  |  | IR | -0.90^***^ | 0.10 | <.001 |
|  |  |  |  |  |  |  |  |  |  | IT | -1.02^***^ | 0.10 | <.001 |
|  |  |  |  |  |  |  |  |  |  | KZ | -0.78^***^ | 0.09 | <.001 |
|  |  |  |  |  |  |  |  |  | RO | CN | -0.39^*^ | 0.12 | .020 |
|  |  |  |  |  |  |  |  |  |  | EG | -0.83^***^ | 0.12 | <.001 |
|  |  |  |  |  |  |  |  |  |  | IR | -0.77^***^ | 0.13 | <.001 |
|  |  |  |  |  |  |  |  |  |  | IT | -0.90^***^ | 0.13 | <.001 |
|  |  |  |  |  |  |  |  |  |  | KZ | -0.65^***^ | 0.12 | <.001 |
| *Note:* **Only significant results were presented at the table.** Goal: 1 – Health (right levels of: blood pressure. cholesterol. body mass. etc.); 2 – Physical fitness. being ‘in shape’; 3 – Company of other people; 4 – Fit. shapely body (beauty. sculpted and firm body); 5 – Wellbeing; 6 – Being physically active and fit according to fashion; 7 – Boosting confidence. gaining appreciation from others; 8 – Pleasure from physical activity; 9 – Escape from everyday life; 10 – Managing stress; 11 – Fulfilling the need for activity; 12 – Promoting PA by setting a behaviour example | | | | | | | | | | | | | |
| *p_bonf_ - p*-value with Bonferroni correction  ^***^ - *p_bonf_* < .001; ^**^ - *p_bonf_* < .01; ^*^ - *p_bonf_* < .05 | | | | | | | | | | | | | |
